# Music preferences as an instrument of emotional self-regulation along the business cycle

**DOI:** 10.1007/s10824-022-09454-7

**Published:** 2022-08-08

**Authors:** Juan de Lucio, Marco Palomeque

**Affiliations:** Economics Department, Universidad de Alcalá, Plaza de San Diego s/n, Alcalá de Henares, 28801 Madrid Spain

**Keywords:** Unemployment, Business cycle, Music consumption, Culture, Wellbeing, Z11, E7

## Abstract

This paper studies the influence of macroeconomic conditions on subjective well-being and music preferences. The macroeconomic cycle exerts an effect on happiness and well-being that consumers counterbalance by modifying music consumption. We use machine learning techniques to make a weekly classification of the top 100 songs of Billboard Hot 100 into positive and negative lyrics over the period 1958–2019. When unemployment is high, society generally prefers more positive songs. Other macroeconomic indicators such as high inflation, high interest rates or low stock market prices also affect musical preferences. These results provide initial evidence regarding the use of cultural consumption to offset business cycle oscillations.

## Introduction

Macroeconomic conditions affect societal consumption, happiness, and wellbeing. People use consumption to offset the negative impact of economic fluctuations. This paper shows that the consumption of cultural services with low production marginal costs, is an instrument to counterbalance the mood impact of aggregate macroeconomic conditions. This analysis sheds light on the relationship between the consumption decisions for cultural services and the wellbeing effects of business cycles.

A recession increases unemployment, reduces income, and modifies expenditure decisions. Consumers postpone the purchase of durables, use more repair services, provide some services on their own and are most likely prone to pursuing pleasure consumption. Interestingly, in this sense, consumption of some low cost high emotional return products increases during economic downturns. In some cases, consumption increases due to a search for hedonic pleasure or mood regulation. This is known as the lipstick effect. There is no evidence of similar effects for cultural services. Although some information exists regarding the “lipstick effects” related with tangible goods (Hamilton et al., 2019; MacDonald & Dildar, 2020), far less is known with respect to cultural consumption and non-priced services (e.g., environmental goods or listening to music on the radio). In the present paper, we try to fill this gap.

From a macroeconomic perspective, the relationship between wellbeing and the business cycle sheds light on the issue of the ‘personal’ costs of cycles and the strategies used by consumers to overcome adverse economic situations. People use music in order to alleviate stress and anxiety as well as boosting their emotional and mental wellbeing.

We elaborate a new dataset with weekly information relating to the 100 most trendy songs and then go on to analyse the lyrics sentiment. We use the mood collected by music consumption as an indicator of how society regulates the mood effects of business cycles. We do not analyse the amount of music consumed, but the sentiment perceived in the songs consumed. We regress the consumption of lyrics sentiments with macroeconomic variables that reflect the short-term economic fluctuations (mainly unemployment, but also inflation, the stock exchange and the interest rate). We find that society reacts to the general business climate by offsetting the negative wellbeing effects of economic cycles with an increase in the cultural consumption that offers positive emotional rewards. This result is robust with respect to the various economic indicators used as well as the different lyrics sentiment measures. As far as we know, this is the first study to show a higher (lower) preference for positive (negative) songs during less favourable macroeconomic periods of time. As the principal result, this has important implications for wellbeing policies. In tandem, it also provides relevant insights with respect to the significant role of culture as a stabilizer of macroeconomic fluctuation effects on well-being.

The remainder of the article proceeds as follows. Section 2 provides a literature framework. Section 3 describes the methodology and database. Sections 4 and 5 presents the empirical results and robustness checks, respectively. The final section concludes.

## Related literature

Our study encompasses various branches of the literature. First, the paper contributes towards an analysis of the relationship between wellbeing and cultural consumption, here specifically using popular music to reveal consumption preferences along the business cycle. A significant section of the literature focuses on cultural consumption and life satisfaction. Grossi et al. (2012) find a correlation between cultural access and psychological well-being. Hand (2018) shows a modest, but significant, effect of arts attendance on happiness. We also know that music is particularly effective in supporting emotional wellbeing and regulating emotions during event-related fluctuations; Saarikallio (2010) evidences emotional self-regulation during certain periods of life. Also, music consumption patterns have been analysed along the biological cycle. Park et al. (2019) and Heggli et al. (2021) describe fluctuations in daily musical preferences which are dependent upon the particular weekday. Bello and Garcia (2021) reveal that digitization has led an upward trend in music consumption diversity. Hanser et al. (2016) show that music is the most important source of consolation as compared with other soothing behaviours; music offers solace in situations of loss and sadness. Gómez-Zapata et al. (2021) concluded that participating in a music education program improves the levels of well-being and the quality of life of the beneficiary population. This paper analyses the relationship between economic conditions and music consumption demonstrating that music is able to smooth out societal emotions along the cycle.

Second, our study relates to an increasing strand of the literature relating to macroeconomic conditions and their effect on well-being and happiness (Deaton, 2012),^1^ (Frey & Stutzer, 2010; Wolfers, 2003) and Mertens and Beblo (2016). The consensus is that macroeconomic fluctuations cause income and wealth shocks and produce stress that impacts on consumption patterns and on well-being. Mainly unemployment but also inflation and adverse financial conditions serve to nurture unhappiness. The present paper shows that society acts countercyclically in order to compensate the wellbeing effects of economic fluctuations.

Third, our paper research explore the Natural Language Processing (NLP) techniques with which to build new indicators capable of following social preferences and mood. This contributes towards the subjective well-being (SWB) literature, see Clark (2018) and Dolan et al. (2008) for reviews of the latter. Likewise, it opens the door to new indicators and approaches for studying SWB applying new data approaches (Bellet & Frijters, 2019). In the music context we highlight the proposal made by Borowiecki (2017) who, using 1400 letters written by three famous composers, measures each individual’s well-being over their lifetime, in an attempt to show that their creativity is linked to negative emotions. As music consumption usually reflects moods, aggregate music consumption collects the overall societal mood. Here, we demonstrate the validity of these indicators.

Additionally, we also know, that the music itself and lyrics, in particular, are the most important aspects of a song in terms of emotional influence, including soothing purposes. Palomeque and de Lucio (2021) show that musical characteristics and lyrics gather different and complementary information. This is the first time that an extensive database of lyrics consumption is built and analysed from a sentimental perspective.

Finally, our paper explains why some types of consumption increase (decrease) during bad (good) times. There are two main explanations for this counterintuitive behaviour. The first approach refers to the idea of inferior goods theory (e.g., consumers buy cheaper goods, pork instead of veal, when income decreases). Music and culture are normal goods, so the latter theory does not fit the inferior good case. Additionally, our work focuses on the hedonic characteristics of lyrics and in this sense, we need to rely upon a theory linked to pleasure seeking music consumption. This brings us to a second theory related with the well-known “lipstick effect.” The “lipstick effect” refers to an increase in the demand and consumption of goods and services characterized by low prices and high personal satisfaction during recessions. This effect relates to the effort of consumers to improve their wellbeing. Two main reasons might explain this behaviour. The first explanation refers to both the search for work-related income (attractiveness to companies) as well as partners (attractiveness to financially stable couples) (Hill et al., 2012). The second possible explanation refers to internal motivations such as: efforts to increase self-esteem (e.g., looking prettier or smarter); efforts to find personal satisfaction through cheap indulgences rather than expensive pleasures; or efforts to use consumption as a morale booster or mood compensator. The preference for positive music is a good example of the last alternative explanation.

**Fig. 1:**
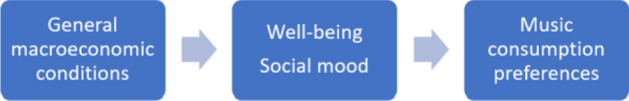
Relational structure. *Source*: Own elaboration

In line with the aforementioned theoretical framework, our empirical model can be represented by Fig. 1. Macroeconomic performance affects societal mood and wellbeing and therefore people tend to adapt their consumption decisions. We use music preferences taking into consideration the sentiment offered by the lyrics of the most consumed songs. We do not expect any effect as regards music preferences on aggregate macroeconomic evolution (e.g., Federal Reserve interest rate decisions are not expected to be determined by songs played by the Rolling Stones). Instead, we infer a causal effect between economic performance and music consumption decisions using culture as a mood stabilizer.

## Methodology and data description

### The song database

Billboard Hot 100 monitors music preferences over time, and as a dataset it is frequently used in music economics (Cameron, 2016).^2^ Billboard Hot 100 commenced in 1958 and it is a reference for other record charts. Billboard Hot 100 encompasses societal preferences with respect to musical characteristics.

A song appears in the record chart if it is one of the top 100 most consumed songs during a particular reference week. The same song can appear during multiple weeks. We use information ranging from the first published week, 4 August 1958, to the last week of 2019.^3^ A total of 28,747 different songs are identified for the 3205 weeks appearing in the database.^4^

The chart provides the title and performer for each song. Using Spotify API, we obtain the valence of each song, which is a variable that, using musical characteristics such as tempo or rhythm, identifies how positive a song is. Valence ranges from 0 to 1, with 1 proving the happiest and 0 the saddest,^5^ with a higher value reflecting the greater happiness of the song according to its musical characteristics. We will also test the effect of macroeconomic variables on valence.

**Fig. 2:**
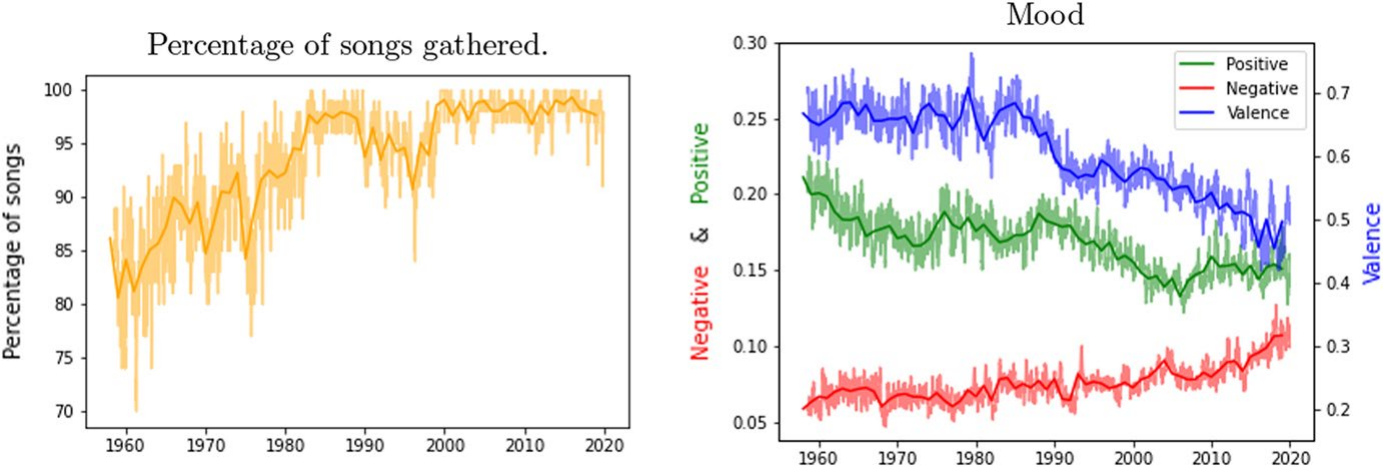
Weekly and yearly time series. *Source*: Own elaboration

Next, we used the title and performer for each song to obtain the lyrics using web-scrapping.^6^ We were able to collect the lyrics for 25,367, 88% of the 28,747 songs that reached the record chart during the period analysed. The left panel of Fig. 2 depicts the evolution of the percentage of top 100 songs collected both in a weekly and yearly basis. Note that most of our study use weekly data. Nevertheless, we also use monthly and annual data when weekly information is not available. We collect almost all the songs that figure on the list. We obtain lesser percentages for the lyrics of the oldest record charts but in general the share is over 80%. The absence of internet tracking for older songs might explain the increasing availability of songs in recent times.^7^

In order to classify whether a song’s lyrics have a positive, neutral or negative message we used a Sentiment Analysis tool called the Valence Aware Dictionary for Sentiment Reasoning (VADER) (Hutto & Gilbert, 2015).^8^ VADER uses a combination of qualitative and quantitative methods to construct a list of lexical features along with their associated sentiment intensity measures. Afterwards, it combines these features with some rules that embody grammatical and syntactical conventions for expressing sentiment intensity. This tool has been used for different purposes, such as analysing the sentiments expressed in Twitter (Elbagir & Yang, 2020) or how positive or negative are student evaluations of teaching (Newman & Joyner, 2018). In our case, we study how positive or negative are the lyrics of those songs that have reached the Billboard Hot 100. VADER offers three coefficients between 0 and 1, one for positiveness, another for negativity and a remaining one for neutrality; the three values sum up to 1. Values close to 1 indicate that the text is mainly influenced by the sentiment that the coefficient measures, while values close to 0 indicate the opposite. We run the Sentimental Analysis for all the songs in our dataset with VADER only functioning with English texts. Most songs are in English, but not all of them. We gathered 78 songs in Spanish, 9 in Korean, and 1 each in Portuguese, Italian, French and Baeggu. Accordingly, we translated the lyrics that are in other languages, using the Python function for Google Translate.

On a weekly basis, we averaged coefficients to develop a summary indicator of the positiveness and negativity of lyrics for each specific week. The right-hand panel of Fig. 2 exhibits the average positive and negative values for each week.

### Macroeconomic variables

In line with the literature we focus upon unemployment but also use three additional macroeconomics variables: inflation, the stock market and the interest rate. We use US data for these indicators as Billboard Hot 100 is based in the US market.

#### Unemployment

The main variable used to measure the socioeconomic situation is usually the unemployment rate. We do not expect composition effects on our estimations, Gardeazabal and Polo-Muro (2021) estimate the effect of unemployment on cultural expenditure and show that unemployment does not affect participation in cultural markets.

Unemployment is considered as the economic variable that most affects an individual’s well-being. Clark and Oswald (1994) find that unemployment has an adverse effect on wellbeing. Ahn et al. (2004) show that the duration of unemployment has a negative impact on individual well-being. It is also well known that unemployment affects mental health. The impact of recessions on mental health refers not only to unemployment but to job insecurity, increased workload and changes in job scope, as well as feelings of helplessness and despair. Employed people also suffer in times of crisis (Modrek & Cullen, 2013).

**Fig. 3:**
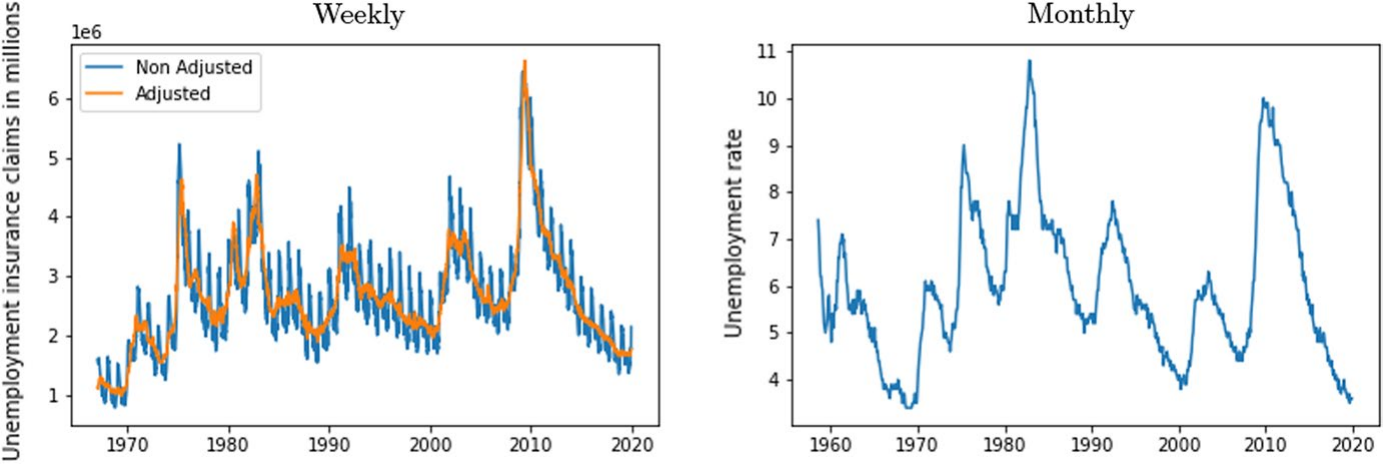
Insurance weekly claims and US unemployment. *Source*: US Department of Labor and Federal Reserve Economic Data

No weekly unemployment data is accessible. Instead, we use the weekly US unemployment insurance claims, available from the US Department of Labor. This data presents strong seasonality, so that seasonal adjusted weekly claims are employed. In this case, we have data available from 1967. The time series are represented in Fig. 3 (both the original and the seasonally adjusted series). In this paper, we also use annual and monthly unemployment data from the start of the Billboard Hot 100 (August 1958) which is available from the Federal Reserve Economic Data (FRED). The right hand panel in Fig. 3 illustrates the monthly time series data.

#### Inflation rate, stock exchange and interest rate

**Fig. 4:**
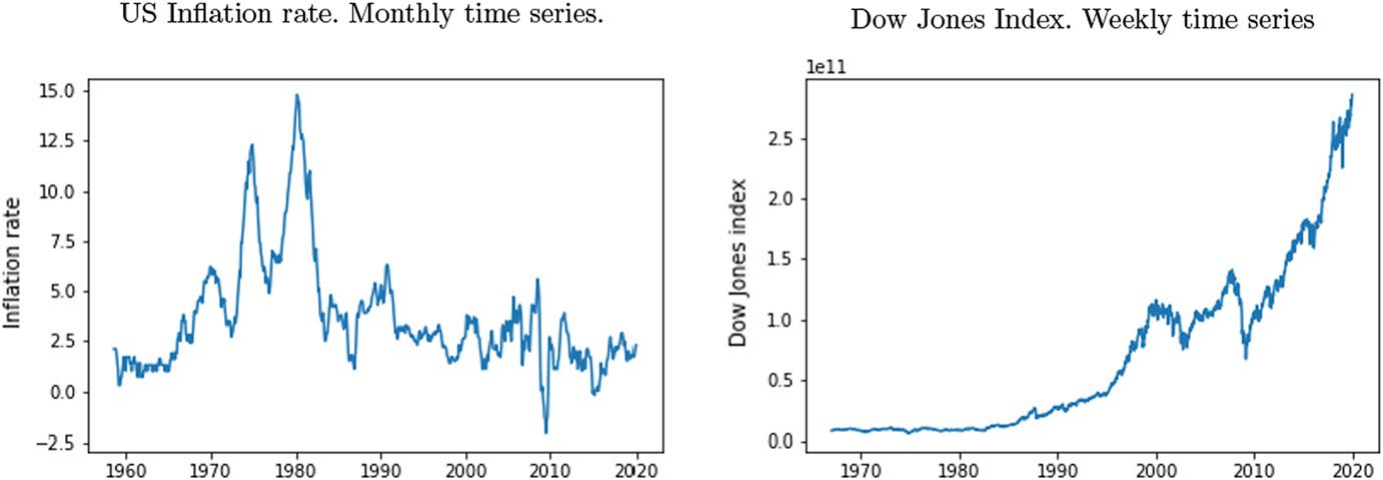
Macroeconomic variables. *Source*: FRED

The literature suggests that a high inflation rate has a negative impact on well-being, Blanchflower et al. (2014), Di Tella et al. (2001). Inflation brings uncertainty, causes reallocation effects and erodes savings. However, the effect seems to be less relevant than the one caused by the unemployment rate. Inflation is not available on a weekly basis, so we use monthly information available from the US Bureau of Labor Statistics database. In the left hand panel of Fig. 4 inflation is depicted from August 1958, (when the Billboard Hot 100 commenced), onwards.

The variable we are going to use to analyse the stock market effect is the Dow Jones index (source: Spanish Ministry of Economy). In this case, the stock market index correlates with prosperity and income, so we expect the results to be the opposite to inflation or unemployment. We expect that positive (negative) lyrics consumption will fall (rise) when the stock exchange reaches higher values.

The right hand panel of Fig. 4 shows the weekly evolution of the index. Graham et al. (2010) reveal a relationship between the happiness of Americans and the Dow Jones index. Cotti et al. (2014) show that a negative monthly Dow Jones index is related to risky consumption, probably coinciding with an emotional offset. Frijters et al. (2015) show that stock market increases lead to modest improvements in life satisfaction and mental health, because of the direct exposure (for young and middle-aged males) or as a leading indicator of employment prospects (for young cohorts). Fernandez-Perez et al. (2020) study music sentiment and stock returns and reveal an inverse relationship between stock exchange prices and valence, which is associated with a mispricing correction.

**Fig. 5:**
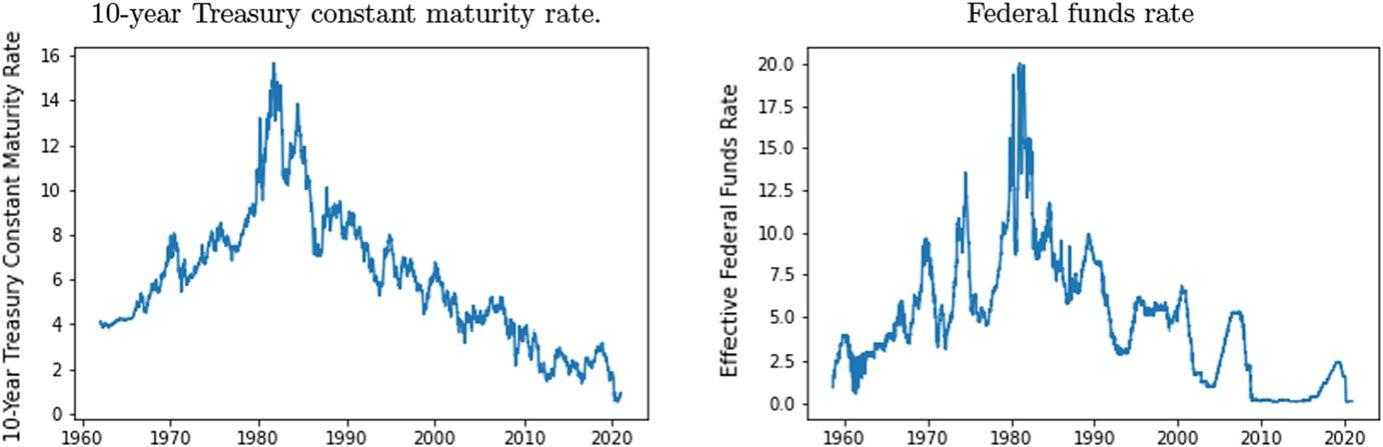
Weekly interest rate. *Source*: FRED and Spanish Ministry of Economy

A high interest rate correlates with high inflation, more expensive mortgages, and lower consumption levels. Therefore, we expect a negative impact of high interest rates on society and a higher desire to consume positive lyrics. For the interest rate, we use two different variables. The two variables employed are the 10-year treasury constant maturity rate and the effective federal funds rate. The first one is more related to expectations, because its effect depends on how people forecast the next 10 years. This allows us to measure whether the effect on music consumption is different when we use variables that measure expectations rather than short-term situations, the latter of wich are more related with the federal funds rate. In Fig. 5 we observe the weekly evolution of interest rates for the period of analysis.

## Empirical results

We try to test whether the economic scenario affects music consumption. We use macroeconomic variables related with the SWB of society to analyse its relationship with societal preferences for music. We regress the mean coefficients of positive, negative and valence, each represented by, $\mathrm{LS}_t$ (the lyrics sentiment), on unemployment insurance weekly claims (and unemployment), inflation, stock exchange variables and interest rates, all included under the general denomination of, $\mathrm{Macro}_t$^9^. We also incorporate a time trend to control the evolution of time dependence on both music consumption and the macroeconomic variables.^10^ Equation 1 defines the standard regressions for the rest of the section.

1 $$\begin{aligned} \mathrm{LS}_t = cte + \beta _{1} \mathrm{Macro}_t + \beta _{2} \mathrm{trend} + \epsilon _t \end{aligned}$$

### Unemployment regressions

The main relationship we wish to explore is between unemployment and the sentiment related to the consumption of music lyrics. Our initial hypothesis considers that music is able to regulate mood, so that positive lyrics are likely to experience a greater consumption when unemployment is high and SWB decreases. To test this, we use Eq. 1 and run the regressions for the positive, negative and valence variables.

In the case of weekly data, the independent variable comprises the weekly unemployment insurance claims from the US Department of Labor. This data has been public since 1967 so the regression will be for the period between the first week of 1967 and the last week of 2019.^11^ A time trend is used to prevent the possibility of a spurious correlation. In this case, we have 2765 observations^12^ (weeks).

In Table 1 we present the result of the weekly regressions. “Positivity” represents the mean coefficient of positive lyrics. “Negativity” stands for the negative lyrics’ coefficient. “Valence” is the mean coefficient of the valence value of each song for each period. The weekly claims are measured in millions of claims, using logarithms. As already mentioned, all regressions incorporate a time trend.

We are unsure whether songs in other languages (e.g., Spanish) have the same effect as songs in the listener’s native language (mostly English in the USA). Probably, some people just listen to these songs because they like their musicality, even though they do not understand the meaning of the lyrics. To control for this, we run a second version of the regressions without resorting to translation. We run regressions with (columns 1 to 3) and without (columns 4 to 6) lyrics translation into English.

**Table 1:** Weekly regressions for weekly claims. *Source*: Own elaboration

Dep. Var.	(1)	(2)	(3)	(4)	(5)	(6)
	With translations			Without translations		
	Positivity	Negativity	Valence	Positivity	Negativity	Valence
Claims(logs)	0.003***	− 0.007***	0.026***	0.003***	− 0.007***	0.027***
	(0.000)	(0.002)	(0.001)	(0.001)	(0.000)	(0.002)
Constant	0.149***	0.160***	0.342***	0.145***	0.164***	0.331***
	(0.011)	(0.007)	(0.028)	(0.011)	(0.007)	(0.028)
Trend	Yes	Yes	Yes	Yes	Yes	Yes
Obs.	2765	2765	2765	2765	2765	2765
$R^2$	0.450	0.593	0.786	0.453	0.595	0.790

*Statistical significance level at 10%

**Statistical significance level at 5%

***Statistical significance at 1%

All coefficients are significant and bear the expected sign. The coefficient is positive for the “Positivity” case, column (1), which confirms our hypothesis: when the unemployment rate is high, the consumption of positive lyrics is also high. We obtain the opposite sign for “Negativity”, column (2).^13^ The valence coefficient, column (3), that measures how happy a song is based on its musical characteristics, also has a positive correlation with the unemployment rate.

We see in Table 1, columns (4) to (6), that the results do not change when we use the lyrics translation. The effect is again significant and with the expected sign.

We now know that, when the economic situation worsens, consumers not only prefer positive lyrics but also happy music, which is what we would have expect according to the “lipstick effect”. These results indicate that when people experience drops in SWB they try to find relief in music. For instance one standard deviation in the weekly claims would increase the positiveness index by about 0.3%, decreasing the negative one by about 0.9% and increasing the valence by about 0.7%.^14^ Accordingly the aforementioned results, suggest that the effect is higher as regards the preferences for negative lyrics, than for the musical characteristics and lower with respect to the preferences for positive lyrics.

### The effect of the business cycle

We check whether the effect depends upon the cyclical situation of the economy. Table 2 provides two different sets of tests. In columns (1) and (2) we analyse the effects when the economy is in a period of downturn. The “Decrease dummy” takes value “1” when the economy is decreasing and “0” otherwise, that is from peak to trough.^15^ “Claims* Dum.Dec” is the result of multiplying the “Decrease dummy” by the unemployment claims of the period. In column (1) we observe a negative sign in the societal preferences for positive lyrics when the economy is in a downturn. When considering the interaction between decreasing economic activity and weekly claims, column (2), the effect of an increase in weekly claims on preferences for positiveness in downturns is smaller, but slightly higher during periods of expansion. This points to a short-term effect that disappears with time. To delve deeper into this idea, in columns (3) to (5) we analyse the length of the business cycle.

**Table 2:** Business cycle length, weekly regressions. *Source*: Own elaboration

Dep. Var.	(1)	(2)	(3)	(4)	(5)
	Positivity	Positivity	Positivity	Positivity	Positivity
Claims (logs)	0.004***	0.004***	0.005***	0.018***	0.019***
	(0.001)	(0.001)	(0.001)	(0.001)	(0.001)
Decrease dummy	− 0.005***				
	(0.001)				
Claims * Dec. Dum.		− 0.003***			
		(0.000)			
Decrease			− 0.002***		− 0.001***
			(0.000)		(0.000)
Growth				0.0004***	0.0004***
				(0.000)	(0.000)
Constant	0.134***	0.133***	0.117***	− 0.082***	− 0.084***
	(0.011)	(0.011)	(0.011)	(0.017)	(0.017)
Date	Yes	Yes	Yes	Yes	Yes
Observations	2765	2765	2765	2765	2765
$R^2$	0.459	0.459	0.467	0.503	0.509

*Statistical significance level at 10%

**Statistical significance level at 5%

***Statistical significance at 1%

“Decrease” is a variable that counts the number of periods during which the economy is experiencing a downturn. That is, the first month after a peak has the value 0,1, and each period afterwards is calculated according to this formula: $\mathrm{down}_t = \mathrm{down}_{(t-1)} + 0,1$ until the economy reaches a trough. When the economy grows, the value is always 0. “Growth” is built the other way around: it counts the number of periods during which the economy is experiencing an upturn and the value is 0 when the economy is contracting.

As mentioned before, what we observe is an immediate effect of unemployment on the consumption of music that counterbalances the effect of downturns. As time passes, the economy and the sentiment adapt to the situation and the effect slowly fades out. This is represented by the negative sign of the decrease variables (the more periods in recession, the less positive is music consumption) and the positive statistical sign of the growth indicator. When both indicators are included simultaneously results do not vary, column (5). The magnitude for the dummy with decreasing economic activity is three times higher than the one observed in recuperation phases, an asymmetric pattern of societal adaptation to the different business cycles phases.

### Additional business cycle variables

Although unemployment seems to be the variable with more impact on SWB we explore other economics variables.^16^ As mentioned in the literature review, the inflation rate can affect the welfare of a society. In Table 3 we show the results of the same regressions (using monthly data) but using the inflation rate instead of the unemployment indicator. For the cases of positive lyrics, column (1), the effect is not significant. However, it is significant for negative lyrics, column (2), and valence, column (3). The effect on valence is positive, meaning that a high interest rate leads to happier music consumption, just as in the case of the weekly unemployment claims. The effect on negative lyrics consumption is the opposite: when the inflation rate increases, the consumption of negative lyrics falls. Even though we lack evidence of people consuming lyrics that are more positive when the interest rate grows, our results appear to suggest that people are less likely to listen to negative lyrics, i.e., they seemingly avoid listening to music which could provoke depression. As mentioned previously, we expected the inflation rate effect to be lower than the unemployment rate effect, this being in line with our hypothesis of not finding significance for all the variables.

**Table 3:** Monthly regressions for inflation rate and weekly for Dow Jones index. *Source*: Own elaboration

Dep. Var.	(1)	(2)	(3)	(4)	(5)	(6)
	Positivity	Negativity	Valence	Positive	Negative	Valence
Inflation	0.009	− 0.063***	0.433***			
	(0.016)	(0.011)	(0.043)			
Dow Jones				− 0.670***	1.035***	− 3.067***
				(0.080)	(0.049)	(0.207)
Constant	0.190***	0.064***	0.680***	0.184***	0.064***	0.705***
	(0.001)	(0.001)	(0.003)	(0.001)	(0.000)	(0.002)
Trend	Yes	Yes	Yes	Yes	Yes	Yes
Observ.	737	737	737	2765	2765	2765
$R^2$	0.566	0.549	0.787	0.461	0.621	0.788

*Statistical significance level at 10%

**Statistical significance level at 5%

***Statistical significance at 1%

**Table 4:** Weekly regressions for interest rate. *Source*: Own elaboration

Dep. Var.	(1)	(2)	(3)	(1)	(2)	(3)
	10-Year treasure			Federal funds		
	Positivity	Negativity	Valence	Positive	Negative	Valence
Interest	0.150***	− 0.027***	0.578***	0.078***	− 0.006	0.352***
	(0.011)	(0.007)	(0.027)	(0.008)	(0.005)	(0.021)
Constant	0.172***	0.060***	0.662***	0.180***	0.057***	0.686***
	(0.001)	(0.001)	(0.003)	(0.001)	(0.001)	(0.003)
Trend	Yes	Yes	Yes	Yes	Yes	Yes
Observations	2765	2765	2765	2765	2765	2765
$R^2$	0.486	0.562	0.805	0.465	0.560	0.793

*Statistical significance level at 10%

**Statistical significance level at 5%

***Statistical significance at 1%

We now return to weekly data. The right-hand panel in Table 3 exhibits the analysis for the weekly stock market indicator (Dow Jones). In this case, we expect opposite signs because it measures a good socioeconomic situation. All the parameters are statistically significant and with the expected sign. In the case of the Dow Jones Index regressions, columns (4) to (6) in Table 3, we find a negative and significant effect for positive lyrics and valence and a positive effect for negative lyrics. Hence, the consumption of positive lyrics decreases as well as the consumption of happy songs (valence). Higher stock market prices increase the consumption of negative lyrics.

The study confirms that situations such as high inflation or low stock market indices which reflect worse socioeconomic circumstances, provoke individuals to search for ways to compensate themselves emotionally in the form of listening to happy music.

The last set of variables studied are the ones related to the interest rate. Table 4 shows that, in both cases, the results obtained coincide with our hypothesis: a worse situation leads to more consumption of happy music as compared with sadder tunes. However, the effect for negative lyrics is only significant in the case of the 10-year treasury constant maturity rate.

## Robustness

Unemployment is the variable through which economic business cycles most clearly affect short-term SWB. We focus the robustness tests on this variable and on the dependent variables proposed in the article. We perform six sets of robustness checks. The first two checks propose modifications in the dependent variable. Then we focus on monthly and yearly data, instead of weekly data. The fourth robustness analysis considers unemployment and inflation indicators simultaneously. The fifth robustness check measures the situation of the labour market with new variables, such us long-term unemployment. Finally, we analyse the impact of the length of the business cycle in a monthly basis.

### Alternative measures of the dependent variable

We build two alternative indicators for the sentiment indicator. For this set of robustness checks we consider only weekly unemployment insurance claims.^17^

**Table 5:** Weekly regressions for seasonally adjusted unemployment. New sentiment indicator. *Source*: Own elaboration

Dep. Var.	(1)	(2)	(3)	(4)	(5)	(6)
	Weighted			Top 10		
	Positivity	Negativity	Valence	Positivity	Negativity	Valence
Claims(logs)	0.006***	− 0.006***	0.016***	0.003*	− 0.007***	0.026***
	(0.002)	(0.001)	(0.006)	(0.001)	(0.000)	(0.002)
Constant	0.104***	0.155***	0.545***	0.149***	0.160***	0.342***
	(0.025)	(0.015)	(0.083)	(0.011)	(0.007)	(0.028)
Trend	Yes	Yes	Yes	Yes	Yes	Yes
Obs.	2765	2765	2765	2765	2765	2765
$R^2$	0.168	0.164	0.408	0.450	0.593	0.786

*Statistical significance level at 10%

**Statistical significance level at 5%

***Statistical significance at 1%

The left hand panel of Table 5 exhibits the results for the weighted indicators (dividing each song’s positive, negative and valence coefficients by the position of the song in the chart). The idea is that the song in the top position is listened to more frequently than the one at the bottom of the list, so that the sentiment of this song might better capture the societal mood. Columns (1) to (3) show that the coefficients remain significant and with the expected sign.

Columns (4) to (6) provide results when the dependent variable is the average of the top 10 songs, instead of the top 100 songs, for each period. Results do not vary in relation with the previous estimations and confirm that society tries to compensate the well-being effects of economic conjuncture through music consumption.

### Alternative positiveness measures

We now build three new indicators of positiveness for the Billboard Hot 100. In this case, the procedure to build the new indicators only provides the degree of positiveness but does not deliver negative sentiment indicators. So, in these robustness checks regressions we only consider the macroeconomic effect on positive sentiment consumption.

First, we use the VADER positive and negative coefficients in order to classify a song as positive. We consider that a song is positive if its positive coefficient is at least twice its negative coefficient. Then, we construct a new index incorporation the percentage of positive songs for each week. Table 6, column (1), presents the estimations using this variable, which maintains the interpretative power of the previous cases.

Second, we calculate the mean positive words per week as the percentage of positive words per total words in the lyrics of the songs listened to that week. The procedure is as follows. We gather a list of 265 positive words^18^ and we counted the times that each one of them appears in the lyrics. Then, we count the total amount of words of each song and finally divide the number of positive words by the total amount of words. Results are also consistent with our previous results, see Table 6, column (2).

Third, the final method we used to measure positiveness in lyrics is TextBlob (Kunal et al., 2018). TextBlob is a tool that works similar to VADER and it offers us only one coefficient, between $-1$ and 1. The text is more negative when closer to $-1$ and more positive when closer to 1. In Table 6 column (3) we see that results are also consistent with the ones presented: when unemployment is high society prefers the consumption of positive lyrics.

**Table 6:** Weekly regressions for seasonally adjusted unemployment. New positiveness indicators. *Source*: Own elaboration

Dep. Var.	(1)	(2)	(3)
	% Positive songs	Mean positive words	TextBlob
	Positivity	Positivity	Positivity
Claims (logs)	0.041***	0.001***	0.018***
	(0.003)	(0.000)	(0.001)
Constant	0.127***	0.036***	− 0.073***
	(0.050)	(0.003)	(0.018)
Trend	Yes	Yes	Yes
Observations	2765	2765	2765
$R^2$	0.633	0.043	0.665

*Statistical significance level at 10%

**Statistical significance level at 5%

***Statistical significance at 1%

### Monthly and yearly data

Our previous results for unemployment are weekly. We now use monthly and annual unemployment indicators. For the annual and monthly regressions, the business cycle variable will be the unemployment rate from the FRED database. The period covers the period from the start of the Billboard Hot 100 (August 1958) to December 2019. We have 62 observations for annual data and 737 for monthly data.

**Table 7:** Monthly and annual regressions. *Source*: Own elaboration

Dep. Var.	(1)	(2)	(3)	(4)	(5)	(6)
	Monthly			Annual		
	Posivity	Negativity	Valence	Posivity	Negativity	Valence
Unemployment	0.147***	− 0.092***	0.483***	0.166**	− 0.102**	0.485**
	(0.027)	(0.018)	(0.076)	(0.072)	(0.049)	(0.219)
Constant	0.182***	0.067***	0.673***	1.738***	− 0.875***	7.187***
	(0.002)	(0.001)	(0.005)	(0.125)	(0.085)	(0.378)
Trend	Yes	Yes	Yes	Yes	Yes	Yes
Observations	737	737	737	62	62	62
$R^2$	0.582	0.544	0.770	0.734	0.686	0.839

*Statistical significance level at 10%

**Statistical significance level at 5%

***Statistical significance at 1%

The monthly estimation, columns (1) to (3), in Table 7 we observe that the results confirm those obtained with the weekly information displaying statistically significant coefficients for all indicators; positive, negative and valence. When unemployment is high society prefers more positive songs and less negative song, also increasing the preference for songs with higher valence. We observe the same behaviour with annual data, see columns (4) to (6), although the significance level is slightly lower, pointing to a short-term relation between music preferences and macroeconomic variables.

### Inflation and unemployment simultaneously

Unemployment and inflation are simultaneously present in different economic models; Phillips curve, Taylor rule, Okun misery index, for instance. We test the performance of both variables simultaneously in determining SWB. Table 8 presents the estimation results. Unemployment maintains signs and significance; the inflation rate loses significance for positive regressions but keeps its sign and significance for negative and valence indicators. This result is in line with (Blanchflower et al., 2014); unemployment seems to depress well-being more than inflation.

**Table 8:** Monthly regressions for inflation and unemployment rates. *Source*: Own elaboration

Variables	(1)	(2)	(3)
	Positivity	Negativity	Valence
Unemployment	1.486***	− 0.766***	3.722***
	(0.277)	(0.181)	(0.727)
Claims(log)			
Inflation	− 0.055	− 0.552***	3.975***
	(0.162)	(0.106)	(0.426)
Constant	0.182***	0.069***	0.660***
	(0.002)	(0.001)	(0.005)
Trend	Yes	Yes	Yes
Observations	737	737	737
$R^2$	0.582	0.560	0.794

*Statistical significance level at 10%

**Statistical significance level at 5%

***Statistical significance at 1%

### Different dimensions of unemployment

In line with our previous results, unemployment appears to have the greatest impact on short-term SWB. This being the case, we explore alternative labour market indicators in Table 9. Column (1) considers the relationship between the preference for positive music lyrics and the duration of unemployment. Our indicator measures the average amount of weeks during which a person seeks employment (in logarithms). As expected, the effect is positive: positive music consumption rises the longer people remain unemployed.

Column (2) considers long term unemployment measured by the percentage of active population that has remained unemployed during at least 27 consecutive weeks. Again, we observe a statistically significant relationship between a negative economic situation for wellbeing and a greater preference for positive lyrics.

Finally, we consider labour force variation,^19^ measured by the monthly change in the labour force (in logarithms). Participation in the labour force depends both on the attractiveness of entering the labour market and on the need to work. In general, the evolution of the labour force has a mild procyclical behaviour (Evans et al., 2018; Tuzemen & Van Zandweghe, 2018) so that we expect a negative sign. When people are more motivated to work, the labour force grows, and consequently society seeks less positive inspiration from music. Column (3) shows that the parameter is not significant.

**Table 9:** Alternative unemployment monthly regressions. *Source*: Own elaboration

Dep. Var.	(1)	(2)	(3)
	Positivity	Positivity	Positivity
Unemp. duration(logs)	0.010***		
	(0.002)		
Long term unemp.		0.002***	
		(0.001)	
Labour force growth (logs)			− 0.001
			(0.001)
Constant	0.167***	0.189***	0.167***
	(0.004)	(0.001)	(0.001)
Trend	Yes	Yes	No
Obs.	737	737	737
$R^2$	0.585	0.576	0.001

*Statistical significance level at 10%

**Statistical significance level at 5%

***Statistical significance at 1%

### Length of business cycle: monthly

In Sect. 4.2, we try to disentangle the different effects of the business cycle phases with weekly data. Here we check the robustness of these results with monthly unemployment.^20^ We observe in Table 10 that results remain robust. Estimates maintain the statistically significant positive sign for the unemployment rate variable and an effect on positive lyrics preferences that decreases with time when the economy is experiencing a downturn and increases when the economy is growing.

**Table 10:** Business cycle length monthly regressions. *Source*: Own elaboration

Dep. Var.	(1)	(2)	(3)	(4)	(5)
	Positivity	Positivity	Positivity	Positivity	Positivity
Unemployment	0.154***	0.161***	0.173***	0.362***	0.355***
	(0.027)	(0.028)	(0.028)	(0.037)	(0.037)
Decrease dummy	− 0.004***				
	(0.001)				
Unemp. * Dec. Dum.		− 0.536***			
		(0.203)			
Decrease			− 0.007***		− 0.003*
			(0.002)		(0.001)
Growth				0.002***	0.001***
				(0.000)	(0.000)
Constant	0.182***	0.181***	0.181***	0.166***	0.167***
	(0.002)	(0.002)	(0.002)	(0.003)	(0.003)
Date	Yes	Yes	Yes	Yes	Yes
Observations	737	737	737	737	737
$R^2$	0.586	0.586	0.593	0.617	0.619

*Statistical significance level at 10%

**Statistical significance level at 5%

***Statistical significance at 1%

## Conclusions

Well-being and the happiness of society are partially driven by macroeconomic variables. During downturns citizens try to cushion the well-being effects induced by a crisis. The effect of a crisis increases goods consumption and produces the already well known “lipstick effect”. In this paper we demonstrate that cultural consumption is also affected by macroeconomic conditions.

Using the Billboard Top 100 list, we show that the music preferred by society, is affected not only by unemployment, but also by inflation, stock market prices and interest rates. When the unemployment rate increases, society well-being decreases, and music preferences are modified. In these situations, people are prone to the consumption of happy songs and positive lyrics instead of negative ones. Results are maintained when using annual, monthly, and weekly time series and are robust to different measurements of the dependent and independent variables.

We conclude that when the macroeconomic situation worsens, the consumption of music changes because people search for happier songs to compensate any bad macroeconomic circumstances. We show that, not only the consumption of songs with positive lyrics is high, but also the consumption of negative lyrics falls. Indeed, this second reaction in the form of a reduction in negative song consumption, is more statistically significant than for the first case. The rise in positive lyrics consumption is less intense when considering weekly insurance claims and is not significant for inflation, whilst the decrease in negative lyrics consumption proves significant for all cases.

Finally, we find that music consumption, in terms of the sentiment of a song depending on its sound factors (valence) works in the same way as lyrics consumption. It brings more robustness to our hypothesis, as the expectation is that listeners want both: positive lyrics and happy music during bad times. SWB is affected in the short term by macroeconomic variables such as unemployment or inflation.

People try to counteract the effects of business cycles on SWB through their consumption decisions, and the present paper specifically shows how society modifies the characteristics of its preferred music. This effect indicates that culture is a stabilizer of mood and consumption, in line with (Tubadji et al., 2015), who find that cultural consumption plays a significant role in correcting social negatives. Society as a whole turns to culture for its SWB regulation. In this sense, from an economic policy point of view, promoting the supply of culture and diversity to guarantee specific societal needs is a first measure of economic policy. Second, access to culture is also an important point to consider. In this study we have analysed a cultural product of common access and with reduced marginal cost (e.g. music listened via the radio). Therefore, policies of access to culture can help citizens to satisfy their needs and more effectively alleviate fluctuations in their SWB. In general, these two proposals, diversity, and access, are conducive to increasing wellbeing and reducing the negative impacts of economic shocks. The recovery of SWB can actually support the recovery of an economy, although, ironically, during recessions it is more common for policy makers to dismiss culturally related policies and funding.

The paper provide evidence about “lipstick effects” on cultural consumption. It also shows the possibilities of NLP techniques in order to create new indicators related to happiness and well-being. These techniques are also useful in analysing consumer preferences with respect to emotional characteristics. Future research should delve further into the foregoing relationships. The amount of music consumed deserves specific research, but new datasets are needed.

## Figures and Tables

**Table 11:**
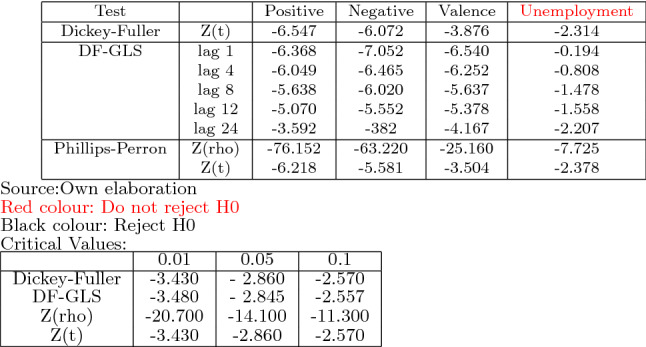
Stationary tests. Main Variables.Test Statistic. *Source*: Own elaboration

**Table 12:**
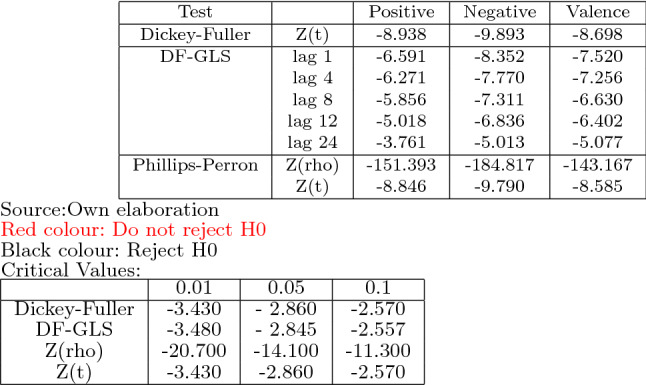
Stationary tests. Employment regressions. Residual test statistics. *Source*: Own elaboration

**Table 13:**
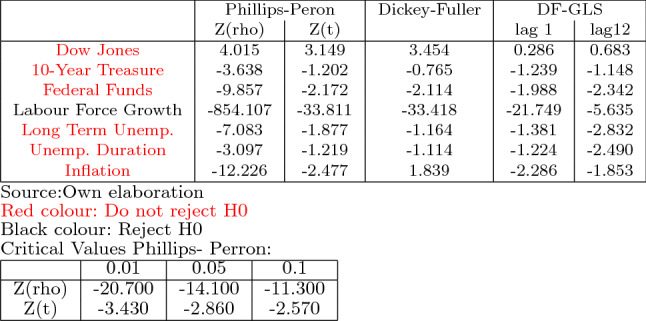
Stationary tests. Phillips–Perron. Other Dependent Variables. *Source*: Own elaboration

## Appendix: Stationary test

Dependent and independent variables and residuals of weekly claims regressions.

See Tables 11, 12 and 13.

## Footnotes

1 Deaton (2012) concludes that subjective well-being monitors short-run levels of anxiety better than the medium-term evolution of the economy.

2 Billboard Hot 100 is a weekly record chart indicating the top 100 songs of the week in US stores, radio plays and, more recently, in digital sources such as YouTube or Spotify.

3 We do not analyse the COVID pandemic exceptional downturn.

4 Although several songs appear and disappear and afterwards reappear some weeks later in the chart, these episodes are infrequent.

5 Valence has recently been used to explain stock exchange variations by Fernandez-Perez et al. (2020).

6 We use three different sources for lyrics, namely, Google, AZ Lyrics and Songs Lyrics. They are among the main lyric websites.

7 The higher the position on the chart the higher probability of collecting the lyrics. We are able to obtain more lyrics for the more successful songs. For a detailed description of the additional insights of the database see Palomeque and de Lucio (2021).

8 These tools for music emotion recognition from lyrics have only recently appeared in the related literature (Agrawal et al., 2021).

9 Independent variables measured in relative terms (eg. unemployment, inflation or interest rates) are expressed in the range [0-1]"

10 We incorporate a trend for non stationary independent variables. Dependent variables and residuals are stationary. See “Appendix” for stationary test.

11 The COVID-19 pandemic needs specific analysis, given that social moods and macroeconomic variables have suffered an unprecedented shock.

12 This data presents seasonality, so seasonal adjusted weekly claims are employed.

13 That is, the correlation between the unemployment rate and negative lyrics consumption is negative. This confirms that the rise in positive lyrics consumption when the unemployment rate increases is attributable to people wanting music to cheer themselves up, as opposed to the case where people listen to songs purely for clearly emotional reasons. Remember that lyrics can be positive, negative or neutral, so both positive and negative lyrics consumption could rise simultaneously. However, we observe how negative lyrics consumption falls with the increase of the unemployment rate, so we can reject this possibility.

14 We use the following reference values: for positive index 0.15, negative index, 0.1, valence 0,5. These reference values are close to the latest values of the corresponding series.

15 We use the NBER Business Cycle Reference Dates https://www.nber.org/research/data/us-business-cycle-expansions-and-contractions, which are determined considering monthly indicators, most of them related with the labour market.

16 There is a debate about the effect of GDP on SWB. Van den Bergh (2009) provides a literature review showing that "GDP per capita (growth) is far from a robust indicator of social welfare". Economic welfare, measured using GDP per capita, differs from social welfare, which has been found not to be connected to GDP levels, Di Tella and MacCulloch (2006) and Di Tella et al. (2001). We exclude GDP and GDP related variables, such us output gap, from this section.

17 The main results do not vary for the other variables: inflation, stock exchange and interest rate.

18 List: absolutely, accepted, acclaimed, accomplish, accomplishment, achievement, action, active, admire, adorable, adventure, affirmative, affluent, agree, agreeable, amazing, angelic, appealing, approve, aptitude, attractive, awesome, beaming, beautiful, believe, beneficial, bliss, bountiful, bounty, brave, bravo, brilliant, bubbly, calm, celebrated, certain, champ, champion, charming, cheery, choice, classic, classical, clean, commend, composed, congratulation, constant, cool, courageous, creative, cute, dazzling, delight, delightful, distinguished, divine, earnest, easy, ecstatic, effective, effervescent, efficient, effortless, electrifying, elegant, enchanting, encouraging, endorsed, energetic, energized, engaging, enthusiastic, essential, esteemed, ethical, excellent, exciting, exquisite, fabulous, fair, familiar, famous, fantastic, favorable, fetching, fine, fitting, flourishing, fortunate, free, fresh, friendly, fun, funny, generous, genius, genuine, giving, glamorous, glowing, good, gorgeous, graceful, great, green, grin, growing, handsome, happy, harmonious, healing, healthy, hearty, heavenly, honest, honorable, honored, hug, idea, ideal, imaginative, imagine, impressive, independent, innovate, innovative, instant, instantaneous, instinctive, intellectual, intelligent, intuitive, inventive, jovial, joy, jubilant, keen, kind, knowing, knowledgeable, laugh, learned, legendary, light, lively, lovely, lucid, lucky, luminous, marvelous, masterful, meaningful, merit, meritorious, miraculous, motivating, moving, natural, nice, novel, now, nurturing, nutritious, okay, one, one-hundred percent, open, optimistic, paradise, perfect, phenomenal, pleasant, pleasurable, plentiful, poised, polished, popular, positive, powerful, prepared, pretty, principled, productive, progress, prominent, protected, proud, quality, quick, quiet, ready, reassuring, refined, refreshing, rejoice, reliable, remarkable, resounding, respected, restored, reward, rewarding, right, robust, safe, satisfactory, secure, seemly, simple, skilled, skillful, smile, soulful, sparkling, special, spirited, spiritual, stirring, stunning, stupendous, success, successful, sunny, super, superb, supporting, surprising, terrific, thorough, thrilling, thriving, tops, tranquil, transformative, transforming, trusting, truthful, unreal, unwavering, up, upbeat, upright, upstanding, valued, vibrant, victorious, victory, vigorous, virtuous, vital, vivacious, wealthy, welcome, well, whole, wholesome, willing, wonderful, wondrous, worthy, wow, yes, yummy, zeal, zealous, Data source: https://www.enchantedlearning.com/wordlist/positivewords.shtml.

19 We have not included a trend in this case because this variable has no unit root.

20 Note that in this robustness control the different dummy variables are built on a monthly basis instead of a weekly one as described in Sect. 4.2.
